# Orofacial Muscle Strength across the Dysarthrias

**DOI:** 10.3390/brainsci12030365

**Published:** 2022-03-10

**Authors:** Heather M. Clark, Joseph R. Duffy, Edythe A. Strand, Holly Hanley, Nancy Pearl Solomon

**Affiliations:** 1 Mayo Clinic, Rochester, MN 55905, USA; jduffy@mayo.edu (J.R.D.); edythestrand@gmail.com (E.A.S.); 2 Department of Communication Sciences and Disorders, Appalachian State University, Boone, NC 28608, USA; hanleyhb@appstate.edu; 3 National Military Audiology and Speech Pathology Center, Walter Reed National Military Medical Center, Bethesda, MD 20814, USA; nancy.p.solomon.civ@mail.mil; 4 Department of Surgery, Uniformed Services University of the Health Sciences, Bethesda, MD 20814, USA

**Keywords:** dysarthria, orofacial strength, assessment

## Abstract

This study compared orofacial muscle strength between normal and dysarthric speakers and across types of dysarthria, and examined correlations between strength and dysarthria severity. Participants included 79 speakers with flaccid, spastic, mixed spastic–flaccid, ataxic, or hypokinetic dysarthria and 33 healthy controls. Maximum pressure generation (P_max_) by the tongue, lips, and cheeks represented strength. P_max_ was lower for speakers with mixed spastic–flaccid dysarthria for all tongue and lip measures, as well as for speakers with flaccid or spastic dysarthria for anterior tongue elevation and lip compression. Anterior tongue elevation and cheek compression tended to be lower than normal for the hypokinetic group. P_max_ did not differ significantly between controls and speakers with ataxic dysarthria on any measure. Correlations were generally weak between dysarthria severity and orofacial weakness but were stronger in the dysarthria groups with more prominent orofacial weakness. The results generally support predictions that orofacial weakness accompanies flaccid and/or spastic dysarthria but not ataxic dysarthria. The findings support including type of dysarthria as a variable of interest when examining orofacial weakness in motor speech disorders.

## 1. Introduction

The dysarthria classification system proposed by Darley, Aronson, and Brown (DAB) [1] has been maintained and refined by contemporary scholars [2,3]. Indeed, it remains the gold standard for distinguishing types of dysarthria based on perceptual speech features. These authors posited that the dysarthria types, while perceptually distinct, arise from specific neuromuscular deficits and can be localized to unique pathways in the nervous system. Flaccid (bulbar) and spastic (pseudobulbar) dysarthrias share the feature of neuromuscular weakness, whereas such “paralysis” is not apparent in hypokinetic (parkinsonian), ataxic (cerebellar), or hyperkinetic (dystonia, chorea) dysarthrias. Similar distinctions were proposed by earlier authors as well [4]. A diverse literature has arisen with the goal of better understanding the neuropathophysiology of dysarthria, with several studies confirming that average orofacial strength in speakers with dysarthria is reduced relative to speakers without dysarthria [5,6,7,8,9,10,11,12,13,14,15,16,17,18,19,20,21]. These studies have typically focused on speakers with a specific neuropathology (e.g., Parkinson disease (PD), stroke, amyotrophic lateral sclerosis (ALS)), with a smaller number of studies examining groups of speakers with dysarthria regardless of etiology or dysarthria type [7,22].

To our knowledge, only one study has explicitly compared orofacial strength measures across patients categorized by dysarthria type according to the DAB scheme. Dworkin and Aronson [7] studied maximum force generation during tongue protrusion and lateralization by speakers with flaccid (*N* = 2), spastic (*N* = 3), ataxic (*N* = 5), hypokinetic (*N* = 1), hyperkinetic (*N* = 1), and mixed dysarthrias (*N* = 6). Although this group of 18 speakers with dysarthria demonstrated significantly lower tongue strength compared to 50 neurologically normal adults, no significant differences in tongue strength were detected among dysarthria types. In some studies, in which dysarthria type was not explicitly reported, it could be predicted from the neurologic diagnosis of the patient group studied. For example, speakers with PD [9,17] would be predicted to exhibit hypokinetic dysarthria, and speakers with muscular diseases such as Pompe disease [16,23], and oculopharyngeal muscular dystrophy (OPMD) [24] would be predicted to exhibit flaccid dysarthria. Studies of orofacial strength in ALS have included patients who were explicitly identified as demonstrating mixed spastic–flaccid dysarthria [6,21] or as demonstrating predominantly flaccid or spastic dysarthria [15,21]. When not stated explicitly [5], the predicted dysarthria type associated with ALS would be expected to be mixed spastic–flaccid.

Most studies examining orofacial strength in dysarthria have reported measures of tongue strength. Some of the earliest studies [6] used force transducers to assess tongue protrusion and lateralization, whereas most recent studies report tongue-elevation (linguapalatal) strength according to maximum pressure (P_max_) generation because of the availability of the Iowa Oral Performance Instrument (IOPI) [25]. A few studies have reported measures of lip and/or cheek strength instead of or in addition to measures of tongue strength [19,20,22]. When more than one structure has been studied, tongue weakness has been more prominent than weakness in other orofacial articulators, but the relationship with dysarthria severity for the various articulators has varied widely across reports [5,14,15,22].

Regardless of the structure tested, studies comparing orofacial strength in patients with dysarthria to that of healthy speakers consistently reported group differences, yet findings have varied in the degree to which the severity of weakness relates to severity of dysarthria or other indices of speech impairment. For example, correlation coefficients between tongue strength and subjective ratings of speech severity have ranged from 0.688 to 0.95 in patients with ALS [5,6,15]. Solomon et al. [22] reported moderate correlations (ranging from 0.455 to 0.524) between various tongue strength measures and speech severity ratings for speakers with varied etiologies. In contrast, studies have reported nonsignificant or weak correlations for speakers with PD [8], OPMD [12] and various other medical diagnoses [7].

The DAB model, which predicts weakness associated with some dysarthrias but not others, may provide a potential explanation for this discrepancy in findings across studies. In the original papers by Darley et al. [1,26] and in the most recent updates of the classification system [2], flaccid, spastic, and mixed spastic–flaccid dysarthrias are explicitly characterized as being related to neuromuscular *weakness*. In contrast, Darley et al. [26] described *limited force of contraction* in hypokinetic dysarthria and *errors in force* in ataxic dysarthria. Indeed, as the DAB model has been refined and is now detailed by Duffy [2], weakness is not considered a hallmark feature of either hypokinetic or ataxic dysarthria. Yet even with these refined predictions, the only published study that directly compared orofacial strength across dysarthria types had a very small sample [7] and has not been replicated with modern methods.

The current study addresses this gap in the literature by asking three research questions: (1) Do participants with dysarthria (PWD) exhibit orofacial weakness compared to control participants without dysarthria? This broad question is addressed as a partial replication of the study by Solomon et al. [22] by examining six tasks involving lingual and facial muscles. (2) Does the degree of orofacial weakness vary across dysarthria types as predicted by the DAB classification system? This question was addressed by comparing all groups to each other and by examining effect sizes of orofacial strength differences for each dysarthria group compared to healthy controls. Finally, (3) is the degree of orofacial weakness associated with the severity of dysarthria? This question was considered exploratory since the dysarthria subgroups were limited in size and range of severity, as determined by expert clinical ratings. Each question was evaluated for each of six orofacial-strength tasks: anterior tongue elevation, posterior tongue elevation, tongue protrusion, tongue lateralization, cheek compression, and lip compression. We hypothesized that lower orofacial strength would be reduced for PWD compared to neurologically normal controls overall, but to a greater degree in the flaccid, spastic, and mixed spastic–flaccid groups than the ataxic and hypokinetic groups. Further, we hypothesized that orofacial weakness and severity of dysarthria would be moderately correlated in the flaccid, spastic, and mixed spastic–flaccid groups.

## 2. Materials and Methods

All subjects gave their informed consent for inclusion before they participated in the study. The study was conducted in accordance with the Declaration of Helsinki, and the protocol was approved by the Institutional Review Boards at Mayo Clinic, Appalachian State University, and Walter Reed National Military Medical Center. The data reported here are part of a larger project examining orofacial muscle strength, muscle tone, speech production, and swallowing function in healthy adults and patients with neurologic disease. Control participants represent a subset of previously reported data [27], which have also been included in related studies [22,28,29].

### 2.1. Participants

PWD were recruited from patients referred to the Speech Pathology Division at the Mayo Clinic (Rochester, Minnesota, USA) for a diagnostic motor speech assessment. Inclusion criteria for the current study were presence of flaccid, spastic, mixed spastic–flaccid, ataxic, or hypokinetic dysarthria as determined by clinical examination by a speech–language pathologist with expertise in the assessment of motor speech disorders (authors JD, ES, or HC) and ability to follow directions to successfully complete the orofacial strength assessment task. Seventy-nine participants met the inclusion criteria; their demographic characteristics are listed in Table 1. The majority of participants reported a duration of dysarthria of 5 years or less. The most common medical diagnosis for this sample was ALS, followed by PD. Only six participants had medical diagnoses that did not reflect a neurodegenerative condition. Additionally, 33 control participants were selected from a database of healthy adults for whom orofacial strength measures were obtained using the same methodology [27]. Because tongue strength varies systematically with age in adults [25,27], all older participants in the database were selected until the mean age closely matched that of the experimental group (Table 1). Individuals who reported history of structural or neurologic impairments impacting speech or swallowing were excluded from the control group. The groups did not differ significantly by age (Kruskal–Wallis rank sum test, *p* = 0.279).

### 2.2. Clinical Motor Speech Assessment

**Figure 1:**
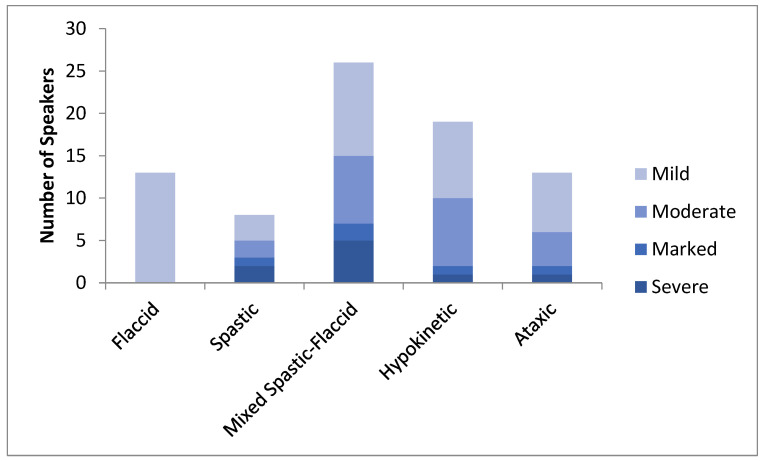
Distribution of participants according to dysarthria severity by dysarthria type.

The diagnosis of dysarthria and its type was based on one expert listener’s (either HC, JC, or ES) perceptual judgments of speech features using a standardized protocol and data-collection form [2]. Tasks included spontaneous speech, oral reading, repetition of sentences, prolonged vowel (/a/), and rapid repetitions of single syllables (alternating motion rates) and series of syllables (sequential motion rates). The hallmark features considered in determining dysarthria type are listed in Table 2 [2,30,31,32,33]. Severity of dysarthria was rated on a 4-point scale (mild, moderate, marked, severe). For reliability purposes, audio recordings of the speech samples for 13 participants (17.5%) were reviewed independently by authors HC and JD a minimum of 4 weeks after the participants’ visits. Original raters were not blinded to patient background, speech complaints, or medical diagnosis, but reliability ratings were judged blindly. Interjudge and intrajudge agreement on differential diagnosis was 85% and 100%, respectively. The two instances of disagreement reflected a categorization of mixed spastic–flaccid dysarthria by the original rater but spastic dysarthria alone by the second rater. Interjudge and intrajudge agreement for severity of dysarthria were each 100% within one scale value and 62% and 80%, respectively, for exact agreement. The original ratings were used in all analyses. Figure 1 illustrates the distribution of severity across dysarthria types.

### 2.3. Orofacial Strength Measures

The measures of tongue, lip, and cheek strength for the PWD were obtained by author HC at the completion of clinical motor speech assessments using the IOPI protocol described previously [22,27,34]. Strength was assessed as the maximum pressure generated (P_max_, in kPa) when participants were instructed to exert maximal effort against an air-filled bulb. Anterior tongue elevation P_max_ was assessed using traditional IOPI procedures [35], with the bulb positioned lengthwise along the hard palate posterior to the central incisors. Posterior tongue elevation P_max_ was obtained with the bulb positioned lengthwise along the hard palate with the distal end of the bulb at the posterior border of the hard palate. Tongue lateralization and protrusion P_max_ measures were obtained with the IOPI bulb affixed to a bulb-holder adapter that was held in place by the teeth and allowed the bulb to be oriented vertically, as illustrated previously [34]. The adapter was positioned between the molars with the bulb facing intraorally for the tongue lateralization measures. Separate measures for lateralization to the right and left were obtained. The adapter was positioned between the upper and lower incisors with the tongue bulb facing intraorally for the tongue protrusion measure.

Measures of cheek P_max_ were obtained with the adapter positioned between the molars as described for tongue lateralization, with the modification that the bulb faced laterally toward the buccal surface. Separate measures for compression by the right and left cheeks were obtained. Lip P_max_ was assessed with the IOPI bulb sandwiched between two wooden tongue blades positioned between the lips at midline (see Figure 1 in [27]). Participants were instructed to lightly place the teeth together and to separate and protrude the lips slightly as the blades were positioned to prevent interdental pressure on the wooden tongue blades and bulb.

Participants were instructed to perform each task with maximum effort. All trials were motivated, with the examiner cheering ‘‘Push, push, push!’’ or ‘‘Squeeze, squeeze, squeeze!’’ P_max_ was recorded for each trial. The best performance of three relatively consistent trials (within 10% of each other) was used as the measure of strength [35]. In rare cases when trials were inadequately consistent, additional trials were performed to meet this criterion. The order of strength tasks was randomized across participants.

### 2.4. Data Analysis

Preliminary analyses included two-sample t-tests for repeated measures to compare measures obtained for right and left tongue lateralization and for right and left cheek compression. Neither measure for tongue lateralization [t (109) = 0.0212; *p* = 0.884] nor cheek compression [t (107) = 2.63; *p* = 0.107] differed for right versus left. Therefore, tongue lateralization and cheek compression measures were averaged for the two sides.

Descriptive statistics (mean and standard error) were calculated for the maximum orofacial strength measure for each task and participant. Pearson correlations examined relationships among the tasks. To examine group differences, strength measures were first subjected to a multivariate analysis of variance (MANOVA) with task as the repeated (within-subject) variable and group as the between-subject variable. Individual Kruskal–Wallis one-way nonparametric tests subsequently tested mean differences in orofacial strength among groups (dysarthria types and healthy control) for each task. An alpha level of 0.008 was adopted to correct for multiple comparisons. Dunn’s nonparametric pairwise follow-up comparisons were conducted using a family-wise alpha level of 0.05. Preplanned Hedges’ *g* effect sizes were performed comparing orofacial strength of each dysarthria group to healthy controls [36]. Effect sizes 0.2 to 0.49 were considered small, 0.5 to 0.79 as medium, and greater than 0.8 as large [37]. Spearman rank correlations were calculated between ratings of dysarthria severity and each of the orofacial strength measures for PWD. Significant correlations (*p* < 0.05) between 0.3 and 0.5, 0.5 and 0.7, and >0.7 were considered weak, moderate, and strong, respectively.

## 3. Results

### 3.1. Missing Data

Data were available from all participants for anterior tongue elevation. At least one participant from one or more groups had missing data for each of the other tasks (Table 3). For posterior tongue elevation, missing data occurred when the participant could not tolerate the posterior position of the bulb. For cheek compression and tongue protrusion and lateralization measures, inadequate dentition precluded stabilizing the bulb adaptor between the lateral or anterior teeth for some participants. One participant was unable to provide valid lip compression measures because of the inability to inhibit the urge to bite the wooden tongue-blade and bulb apparatus rather than squeeze it between the lips.

### 3.2. Group and Task Comparisons

**Figure 2:**
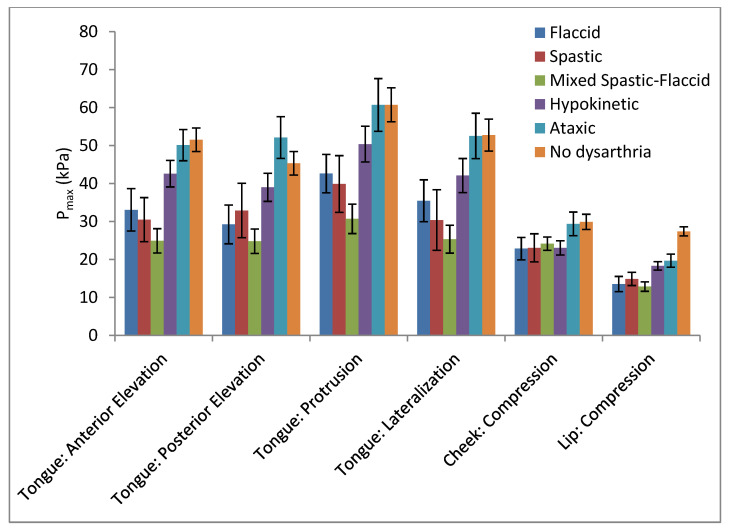
Orofacial strength measures (P_max_, in kPa) averaged for each participant group (five dysarthria types and one control group). Error bars reflect +/− standard error.

Summary statistics and results from inferential statistical analyses for P_max_ across groups for each task are listed in Table 3 and illustrated in Figure 2. To address the first research question, all PWD were combined for comparison to the control group. The experimental group demonstrated significantly lower orofacial strength when data were collapsed for task [*F* (1, 98) = 16.07; *p* < 0.0001]. When collapsed for group, tasks differed significantly such that the facial muscles (cheek, lip) yielded lower P_max_ than the tongue, with tongue protrusion demonstrating the largest P_max_ results compared to the remaining tongue-strength tasks [*F* (2.39, 234.88) = 38.63; *p* < 0.0001]. The interaction between the two groups (PWD and normal) and the six tasks was not significant [*F* (2.39, 24) = 234.88; *p* < 0.0677]. Correlations among tasks are listed in Table 4. Pearson correlation coefficients among the various tongue-strength tasks ranged from 0.7847 to 0.9048. Lip and cheek compression correlated moderately (*r* = 0.5284). Correlations between tongue and facial tasks ranged from 0.3995 to 0.6249. All correlations were statistically significant (*p* < 0.05).

The experimental group was divided according to type of dysarthria to address the second research question. Initial analyses revealed a significant main effect of group [*F* (5, 94) = 8.52; *p* < 0.0001]. As before, the main effect for this task was statistically significant [*F* (5, 90) = 43.58; *p* < 0.0001], but the interaction between the group and task was also significant [Wilkes Lambda (25, 335.84) = 1.95; *p* < 0.004]. Therefore, independent nonparametric group comparisons were completed for each task, for which Welch’s Test for equal variance was significant (*p* < 0.005) for all measures except cheek compression.

Significant main effects for group were observed for each of the orofacial-strength tasks [anterior tongue elevation: *Χ*^2^ (5) = 33.38, *p* < 0.0001; posterior tongue elevation: *Χ*^2^ (5) = 25.19, *p* < 0.0001; tongue protrusion: *Χ*^2^ (5) = 25.28, *p* < 0.0001; tongue lateralization: *Χ*^2^ (5) = 25.12, *p* < 0.0001; lip compression *Χ*^2^ (5) = 53.65, *p* = 0.0001] except cheek compression [*F* (5, 102) = 2.11, *p* = 0.07]. Specific group comparisons and effect sizes are indicated in Table 3. P_max_ for anterior tongue elevation was significantly greater for controls compared to the flaccid, spastic, and mixed spastic–flaccid groups and for the ataxic group compared to the mixed spastic–flaccid group. Comparisons of P_max_ for anterior-tongue elevation between each dysarthria group and the healthy control group revealed large effect sizes for the flaccid, spastic, and mixed spastic–flaccid groups and a medium effect size for the hypokinetic group.

For posterior tongue-elevation, tongue protrusion, and tongue lateralization, the control group and the ataxic group demonstrated higher P_max_ values than the mixed spastic–flaccid group; no other group differences were statistically significant. Effect sizes for posterior tongue elevation were large for the flaccid and mixed spastic–flaccid dysarthria groups, medium for the spastic group, and small for the hypokinetic group. A small effect size was also observed for the ataxic group but in the opposite direction as the other groups: The ataxic group had higher mean posterior tongue elevation strength compared to the control group. For tongue protrusion and lateralization, effect sizes were large for the spastic and mixed spastic–flaccid dysarthria groups, medium for the flaccid group, and small for the hypokinetic group.

Lip compression P_max_ was highest for the control group compared to all groups with dysarthria except the ataxic group, which did not differ significantly from any other groups. Large effect sizes were observed for each of the dysarthria groups. Medium effect sizes for cheek compression were noted for each of the dysarthria groups except the ataxic group despite the lack of significant group effects.

### 3.3. Relationship of Orofacial Strength to Dysarthria Severity

To explore for associations of dysarthria severity with orofacial strength measures for the third research question, Spearman correlations were calculated for each of the orofacial-strength tasks using data from the PWD (Table 4). Correlations between tongue strength and dysarthria severity were weak, ranging from *r* = −0.2708 for posterior elevation to *r* = −0.4474 for protrusion. Facial strength was weakly correlated with dysarthria severity, with *r* = −0.2458 for cheek compression and *r* = −0.3321 for lip compression. All correlations were statistically significant at *p* < 0.05.

**Figure 3:**
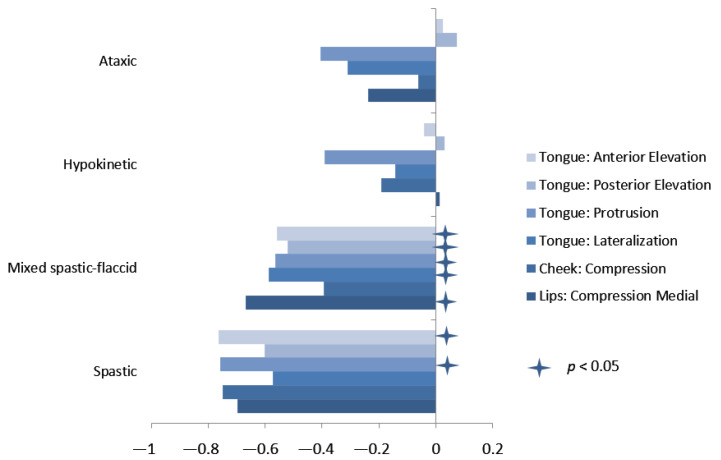
Correlations between strength measures and dysarthria severity by group.

Spearman correlations were also conducted separately for each dysarthria group except for the flaccid group because all of its members had the same level of severity (Figure 3). Correlations were moderate to strong for the spastic group, which was also the smallest group, ranging from *r* = −0.5714 to *r* = −0.7629; among these, the correlations were statistically significant for anterior tongue elevation and tongue protrusion (*p* < 0.05). Correlations for the mixed spastic–flaccid group were weak to moderate, ranging from *r* = −0.3945 to *r* = −0.6692, but because this group was larger, the correlations were significant for all measures except cheek compression (*p* < 0.05). For the ataxic and hypokinetic dysarthria groups, dysarthria severity correlated weakly with tongue protrusion P_max_ (*r* = −0.3913 and *r* = −0.4045, respectively).

## 4. Discussion

This study systematically assessed orofacial strength across dysarthria types to test hypotheses inherent to the DAB dysarthria classification scheme that neuromuscular orofacial weakness is associated with dysarthrias classified as flaccid and/or spastic but not ataxic or hypokinetic.

### 4.1. Orofacial Strength in Speakers with and without Dysarthria

**Figure 4:**
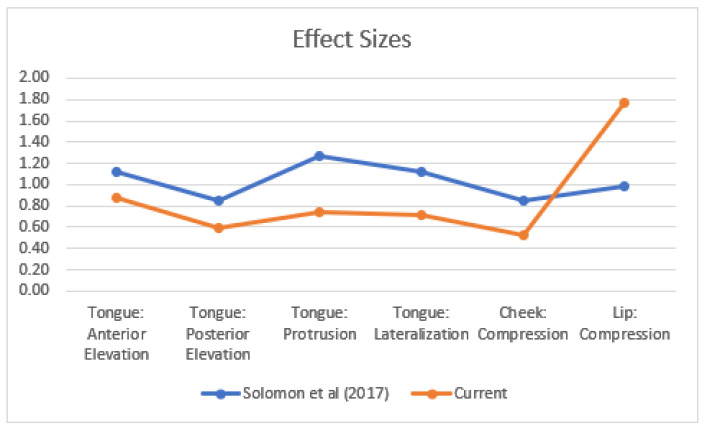
Effect sizes for the individual orofacial strength tasks for PWD from Solomon et al. [22] and the current study.

As predicted, and consistent with a rich literature, a significant group effect was detected for orofacial strength when comparing PWD and healthy age-matched controls. This study replicates the findings of Solomon et al. [22], which also demonstrated significantly lower P_max_ on the same tasks included here in a group of PWD of multiple etiologies. The PWD groups’ results were highly consistent across the two unique PWD participant pools as indicated by effect sizes from each study for the various tasks (Figure 4).

The results for the healthy age-matched control group are consistent with those reported in the parent data set [27] and with larger systematic reviews of normative data for orofacial strength [25]. However, strength measures were lower than the subgroup of controls included in a later study [22], especially for measures of tongue lateralization and protrusion. Furthermore, tongue-strength results reported for the current cohort of PWD, when grouped, were slightly lower than the means reported by Solomon et al. [22], but well within the reported standard deviations. The generally lower P_max_ results in the current study could be attributed in part to the use of a gauze cover on the IOPI bulb to prevent slippage for most of the participants in the 2017 study compared to the use of a bare bulb in the current study. Solomon and Clark [29] directly compared these techniques for tongue-strength tasks and found that the use of gauze as an anti-slip precaution yielded significantly higher P_max_ values for tongue lateralization and protrusion but not for anterior or posterior tongue elevation in healthy adults. The current findings are consistent with reports of reduced anterior tongue strength [5,6,7,10,11,12,13,15,16,18,38] and reduced lip strength in PWD [13,14,18,20,38].

### 4.2. Orofacial Strength across Dysarthria Types

A primary aim of this study was to explore whether orofacial weakness varies across dysarthria types as predicted by the DAB classification system or is similar across types as reported by Dworkin and Aronson [7]. The results should not be interpreted to imply causality between orofacial strength and dysarthria type but rather to explore variables that may be related to a common underlying pathophysiology.

As evidenced by the significant group-by-task interaction, group differences in orofacial strength were not equivalent across the six orofacial strength tasks. Even so, some consistencies were observed. First, speakers with ataxic dysarthria did not differ from controls on any orofacial strength measure. Second, speakers with mixed spastic–flaccid dysarthria demonstrated significantly lower orofacial strength compared to controls and speakers with ataxic dysarthria for every measure except cheek compression. Finally, speakers with hypokinetic dysarthria tended to but did not demonstrate significantly different orofacial strength from any other group, except for lip compression, which was significantly lower when compared to controls and speakers with ataxic dysarthria.

In addition to pairwise comparisons, effect sizes were examined to differentiate possible patterns of weakness between each of the dysarthria types and the control group. Medium or large effect sizes were observed for flaccid, spastic, and mixed spastic–flaccid dysarthria for all tasks in the expected direction (PWD weaker than controls). In contrast, the groups with ataxic and hypokinetic dysarthria demonstrated few significant differences when compared to controls, although some differences had small to large effect sizes, and one (posterior-tongue elevation for the ataxic group) showed an unexpected small effect size such that the control group had lower P_max_ values.

The current findings generally support the hypotheses set forth by DAB. Namely, flaccid and spastic dysarthrias, localizing to the upper motor neuron pathways, lower motor neurons, neuromuscular junction, and/or the muscle itself, are associated with neuromuscular weakness, whereas ataxic dysarthria, localizing to the cerebellar control circuit, is not. Findings for the hypokinetic group, localizing to the basal ganglia control circuit, were hypothesized to be within normal limits, but the present results trended towards reductions in orofacial strength. This conclusion is consistent with a meta-analysis of previous literature [39] that reveals inconsistencies due to sampling differences, including disease severity. Pitts et al. [39] concluded that approximately one-third of people with PD (and perhaps hypokinetic dysarthria) would be expected to demonstrate reduced tongue anterior-elevation strength.

It is worth noting that although rigidity in the perioral system has been associated with reduced lip range of motion in people with PD [40], we are aware of no evidence that rigidity affects orofacial strength. In fact, rigidity and weakness have not been associated in general for people with PD, although both problems do occur [41]. Similarly, ataxic hypotonia [42] is a characteristic of muscle at rest, distinct from the ability of muscle to create force from contraction. Therefore, we would not have predicted hypotonia to influence orofacial strength measures, should it manifest systematically in dysarthria, which has yet to be established [28].

Although the DAB model does not predict that the weakness profile of various dysarthrias will vary across orofacial muscle groups, most reports indicate that weakness associated with dysarthria is most prominent in the tongue [5,14,15,22,38]. Such weakness could impact articulatory precision, although the association is far from clear. In the current study, anterior tongue elevation strength differed the most across the groups, with statistically significant differences separating controls and speakers with ataxic dysarthria from all other groups, and speakers with mixed spastic–flaccid dysarthria from all other groups. The remaining tongue strength measures detected only the most dramatic weakness in the mixed spastic–flaccid dysarthria group. Cheek compression strength did not differ among any of the groups. Lip compression strength in the healthy controls differed from all the groups with dysarthria except ataxic dysarthria, all with large effect sizes.

The prominence of tongue weakness in flaccid, spastic, and mixed flaccid–spastic dysarthria may be multifactorial. First, cortical motor representation for the tongue is relatively large and therefore may have a greater probability of being affected by a cortical stroke or other brain injury [43,44]. Second, given the tongue’s nature as a muscular hydrostat rather a structure moved by fibers contracting around a joint, it may decline more rapidly in its ability to generate high forces compared to other orofacial muscle groups given similar levels of neurologic impairment [45,46]. Third, the impact of tongue weakness on speech may be amplified given the forces needed to move a muscular hydrostat at high speeds [47]. Finally, it is intuitive given the important contribution of articulatory precision for intelligibility that the speech mechanism would be sensitive to disruption of tongue function. However, studies have failed to reveal the anticipated relationships between weakness and intelligibility, as elaborated below [12,22].

### 4.3. Relationship of Severity of Weakness to Severity of Dysarthria

Although reports of orofacial weakness in PWD compared to healthy controls are quite consistent, findings have varied widely in the degree to which the severity of weakness relates to perceptually judged severity of dysarthria or other indices of speech impairment. This study tested the prediction that the strength of correlation between dysarthria severity and orofacial weakness would vary across dysarthria types. The severities of flaccid, spastic, and mixed spastic–flaccid dysarthria were expected to correlate comparatively strongly with orofacial strength.

In the current sample, correlations for flaccid dysarthria could not be calculated because the speakers all exhibited the same severity of dysarthria. Within this group of speakers with mild flaccid dysarthria, variability of tongue strength was as large as that observed for the group of speakers with mild to severe spastic dysarthria. Apparently, the speakers with flaccid dysarthria were able to speak relatively clearly despite some having markedly abnormal tongue weakness, perhaps a testament to their ability to compensate for their deficits with other more intact articulatory structures and functions. While previous literature has not examined orofacial strength specifically for flaccid dysarthria, a small number of studies have included patient groups for whom flaccid dysarthria would be the predicted motor speech disorder. Neel et al. [12] reported weak correlations (below *r* = −0.4) between tongue strength and performance on speech tasks for speakers with OPMD. However, it should be noted that the group of speakers in that sample did not demonstrate dysarthria, in that they did not differ statistically from healthy controls with respect to listener ratings of intelligibility, stress, intonation, rate, articulatory precision, voice quality, or nasality. Jones et al. [16] did not report correlations between dysarthria severity and tongue strength in speakers with Pompe disease, but they did detect statistically significant differences in tongue strength between groups differentiated by dysarthria severity. Further research including participants with differentially diagnosed flaccid dysarthria of varying severity is needed to characterize the relationship between dysarthria severity and orofacial weakness in this group of speakers.

The correlation between weakness and dysarthria severity for the spastic and spastic–flaccid dysarthria groups in the current study ranged from *r* = −0.40 to *r* = −0.78 across tasks. All tasks correlated significantly except cheek compression strength for the mixed spastic–flaccid dysarthria. Although cheek weakness was confirmed in the mixed group, it apparently is less closely related to the severity of dysarthria than tongue and lip weakness. Anterior tongue elevation and tongue protrusion weakness correlated significantly with dysarthria severity in the spastic dysarthria group. No literature was identified that expressly examined spastic dysarthria. Studies that are most appropriate for comparison of both the spastic and mixed spastic–flaccid groups are those including speakers with ALS, who would be presumed to demonstrate spastic, flaccid, or mixed spastic–flaccid dysarthria. These studies reported correlations ranging from *r* = −0.69 to *r* = −0.95 [5,15,48]. The weaker correlations found in the current sample were generally observed for measures that are not typically included in this literature (e.g., tongue lateralization, cheek compression) and may also reflect a greater heterogeneity in medical diagnoses.

No significant correlations were found between any orofacial strength task and dysarthria severity for ataxic and hypokinetic dysarthria. Specifically, correlation coefficients were essentially nil between anterior tongue elevation and dysarthria severity for these groups. No studies were identified that reported correlations specifically for ataxic dysarthria for comparison. The data for hypokinetic dysarthria can be considered in the context of studies with speakers who have PD. Solomon et al. [9] reported a weak, nonsignificant correlation coefficient between anterior tongue elevation and overall speech severity during a spontaneous speech task (*r_S_* = −0.230) for 16 speakers with PD. In a metanalysis involving 35 speakers with PD, Solomon et al. also reported a similar association (*r_S_* = −0.256) between these variables for a picture description task. The differences between these studies may be related to differences in variability for severity; all but two participants in each of these groups in the present study were judged to have mild or moderate dysarthria. There were also differences in methodology for determining dysarthria severity, such that the current study used ratings based on in-person clinical assessments whereas the previous study used ratings of recordings from specific speech tasks with listeners blinded to patient information.

Taken together, the current findings generally support the prediction that the strength of relationship between orofacial weakness and dysarthria severity varies across dysarthria type as implied by the DAB dysarthria classification system. This may explain some of the variability in findings across studies regarding correlations between weakness and dysarthria severity and suggests that dysarthria type is an important participant characteristic to be described in studies examining orofacial strength.

### 4.4. Study Limitations

As this study was conducted within a clinical setting using a convenience sample, several limitations are acknowledged. First, a larger sample size with appropriate representation across the full range of severity across each type of dysarthria is needed to confirm the pattern of findings observed in the current sample. This is particularly important given that flaccid dysarthria is the dysarthria type for which weakness is considered the primary underlying impairment. The extent of the impairment is determined in part by whether the flaccidity is focal or diffuse. In the current group of participants with flaccid dysarthria, pathophysiology was diffuse and thereby affected more than a single cranial nerve/muscle group. Second, diagnoses and severities of dysarthria were based on one clinician’s evaluation [2,49,50], albeit from a selection of exceptionally experienced clinicians using standardized procedures; concern about this is mitigated by good interjudge reliability. Third, the present study did not include participants with hyperkinetic dysarthria because adequate numbers of patients with relevant subtypes of hyperkinetic dysarthria (e.g., oromandibular dystonia, dyskinesia, chorea) were not available during the data collection period. Although muscular weakness is not considered to underlie hyperkinetic dysarthrias, orofacial muscle strengthening exercises are sometimes implemented during therapy to manage dysphagia for patients with involuntary movements [51], so studying this population could be elucidating despite the challenges of measuring orofacial muscle strength in the presence of involuntary movements. Fourth, there was no variability within the flaccid dysarthria group and little variability within the hypokinetic and ataxic groups in terms of severity, so correlations between dysarthria severity and orofacial strength within these groups either could not be examined or should be interpreted with caution. Finally, although sex was not included as a design variable in this study, the majority of participants were male, especially in the control group. This is not considered a major weakness because the parent study that provided the control data did not find a significant sex difference for tongue strength [27]. However, a meta-analysis of 12 studies revealed higher P_max_ values on tongue-elevation tasks for healthy men versus women [52]. Regarding facial strength, sex differences were documented in the parent study. Therefore, comparisons to the control group should be interpreted with caution for facial-strength results.

### 4.5. Clinical Implications

The results of this study provide some basis for including orofacial muscle strength assessment during a motor speech evaluation. Although the results are not specific to each dysarthria type and are therefore not predictive of type, they could contribute supportive evidence for upper motor neuron or lower motor neuron disorders as compared to disorders deriving from basal ganglia or cerebellar control circuits. Furthermore, documenting orofacial muscle strength over time may be clinically relevant for progressive neurologic diseases, as well as for tracking natural or therapy-related recovery.

Clearly, dysarthria type is not determined by impairments solely in the orofacial system. In fact, salient features of most dysarthrias involve respiratory, phonatory, resonatory, and prosodic characteristics. Thus, these results should not be taken to imply that the neuromuscular impairments associated with the articulatory system are of greater relevance for differential diagnosis than those of the other speech subsystems.

### 4.6. Future Directions

Future research that includes larger samples for each of the dysarthria types, including hyperkinetic, and wider ranges of dysarthria severity so that it is equally distributed across types could expand upon the exploratory analyses conducted here. Furthermore, studies that assess weakness across the speech subsystems may reveal underlying impairments that contribute to the greater range of perceptual features that lead to a dysarthria diagnosis. Beyond assessment studies, research is needed to associate the functional impacts of orofacial muscular weakness. The current study examined maximum performance of the orofacial muscle groups rather than performance during functional, submaximal tasks such as speech or swallowing. Often, reduced maximal capacity does not impinge on the forces required during submaximal functional activities, but it does reduce the functional reserve of strength, possibly leading to fatigue. Research focusing on oromotor kinematics and dynamics during dysarthric speech [21,53] may be more relevant to defining the effects of various pathologies associated with the dysarthria types on dysarthria severity. Therefore, it is important that future studies on orofacial muscle function specify type of dysarthria in addition to disease or medical diagnosis.

## 5. Conclusions

The DAB classification system posits that weakness accompanies some but not all dysarthria types. This study mostly replicated previous findings that PWD demonstrate orofacial weakness overall [7,22], but it revealed differences across five types of dysarthria (flaccid, spastic, mixed spastic–flaccid, hypokinetic, ataxic) that were not detected by a small previous study based on two tongue-strength measures [7]. The differences identified support the predictions of the DAB model that neuromuscular weakness underlies the speech impairments observed with flaccid and/or spastic dysarthria but not ataxic dysarthria. Although not a hallmark feature of hypokinetic dysarthria within the DAB model, orofacial weakness may be evident in this group as well. For the spastic and spastic–flaccid dysarthria groups, which had significantly weaker orofacial muscles and a greater range of dysarthria severity than the other groups, correlations were moderately strong between orofacial strength and the severity of the dysarthria.

## Figures and Tables

**Table 1:** Participant Demographics.

Dysarthria Type	*N*	Sex (F:M)	Mean Age (SD)	Medical Diagnoses (*N*)	Mean Time Post-Onset (Months)
Flaccid	13	5:8	64.4 (22.8)	ALS (6) Myasthenia gravis (1) Myopathy (1) Congenital neuromuscular disorder (2) Neoplasm (3)	27.2
Spastic	8	4:4	64.9 (8.23)	ALS (6) Multiple sclerosis (1) Parkinson plus (1)	25.0
Mixed Spastic–flaccid	26	13:13	56.7 (12.4)	ALS (26)	38.9
Hypokinetic	19	2:17	64.3 (9.8)	Parkinson disease (14) Parkinson plus (2) Stroke (1) Unknown (2)	38.4
Ataxic	13	5:8	54.07 (13.6)	Cerebellar disease (8) Parkinson plus (2) Multiple sclerosis (1) Other/Unknown (2)	29.6
Combined Groups	79	29:50	58.8 (14.3) Range 18–83		37.3
Healthy Controls	33	2:31	59.8 (19.8) Range 25–89		n/a

SD = standard deviation.

**Table 2:** Hallmark Features Considered in Differential Diagnosis of Dysarthria [2,30,31,32,33].

Dysarthria Type	Features	
Flaccid	Normal or slow rate Hypernasality Breathiness	Short phrases Articulatory imprecision
Spastic	Slow rate Strained vocal quality Monopitch	Monoloudness Articulatory imprecision Slow and regular AMRs
Mixed Spastic–Flaccid	Slow rate Strained vocal quality Vocal flutter Monopitch	Monoloudness Hypernasality Articulatory imprecision Slow and regular AMRs
Hypokinetic	Rapid rate and/or short rushes of speech Reduced loudness Monopitch	Monoloudness Articulatory imprecision Rapid and blurred AMRs
Ataxic	Slow or normal rate Excess and equal stress Irregular articulatory breakdowns	Telescoping of syllables Irregular AMRs

**Table 3:** Summary Statistics for P_max_ (in kPa) Across Participant Groups for Each Task.

	Flaccid	Spastic	Mixed Spastic–Flaccid	Hypokinetic	Ataxic	Controls (No Dysarthria)
**Tongue: Anterior Elevation**						
# of Missing Data Points						
Mean	33.1 ^B^	30.5 ^B^	24.9 ^B,C^	42.6	50.1 ^A,B^	51.5 ^A^
Standard Deviation	(20.3)	(16.4)	(16.4)	(15.4)	(14.7)	(17.6)
Hedges’ *g*	1.0	1.2	1.55	0.53	0.08	
**Tongue: Posterior Elevation**						
# of Missing Data Points		1	1		1	1
Mean	29.2	32.9	24.8 ^B^	39.0	52.1 ^A^	45.3 ^A^
Standard Deviation	(18.5)	(19.0)	(15.7)	(16.1)	(19.3)	(18.0)
Hedges’ *g*	0.89	0.69	1.21	0.36	0 *.* 4	
**Tongue: Protrusion**						
# of Missing Data Points			1	1		
Mean	42.6	39.9	30.7 ^B^	50.4	60.7 ^A^	60.7 ^A^
Standard Deviation	(18.2)	(21.2)	(19.3)	(20.1)	(25.1)	(25.7)
Hedges’ *g*	0.76	0.84	1.30	0.43	0.00	
**Tongue: Lateralization**						
# of Missing Data Points						2
Mean	35.5	30.4	25.3 ^B^	42.1	52.5 ^A^	52.8 ^A^
Standard Deviation	(19.9)	(22.6)	(18.7)	(19.6)	(21.6)	(23.4)
Hedges’ *g*	0.77	0.96	1.04	0.48	0.01	
**Cheek: Compression**						
# of Missing Data Points			2	2		2
Mean	22.8	23.1	24.2	23.0	29.4	29.9
Standard Deviation	(10.7)	(10.4)	(8.7)	(7.9)	(11.2)	(11.3)
Hedges’ *g*	0.63	0.62	0.56	0.67	0.04	
**Lip: Compression**						
# of Missing Data Points					1	
Mean	13.5 ^B^	14.8 ^B^	12.8 ^B^	18.3 ^B^	19.7 ^A,B^	27.4 ^A^
Standard Deviation	(7.2)	(4.9)	(6.3)	(4.9)	(6.0)	(6.9)
Hedges’ *g*	1.98	1.89	2.19	1.45	1.15	

Results designated by different letters differ significantly (*p* < 0.05); P_max_: Maximum pressure generated against an air-filled bulb across three maximum-effort trials; kPa: kilopascals; #: number; Effect sizes (Hedges’ *g*) indicated by color: large (red), medium (purple), small (green). Hedges’ *g* was compared to the control group.

**Table 4:** Pearson Correlations Among Orofacial Strength Measures for All Participants (*N* = 112), and Spearman Correlations Between Orofacial Strength Measures and Dysarthria Severity for Participants in the Experimental Groups (*N* = 79).

	Tongue: Anterior Elevation	Tongue: Posterior Elevation	Tongue: Protrusion	Tongue: Lateralization	Cheek: Compression	Lips: Compression
Tongue: Anterior Elevation		0.9042	0.7844	0.8448	0.5702	0.4805
Tongue: Posterior Elevation			0.8058	0.8626	0.5535	0.4004
Tongue: Protrusion				0.8874	0.6244	0.4964
Tongue: Lateralization					0.5573	0.4964
Cheek: Compression						0.5288
Dysarthria Severity	−0.3238	−0.2708	−0.4474	−0.3900	−0.2458	−0.3321

All correlations *p* < 0.05.

## Data Availability

Data sharing not applicable. Data involve protected health information.
